# Tissue-Specific Detection of Autophagy and the Related Metabolites in *Bombyx mori*

**DOI:** 10.3390/insects17090949

**Published:** 2026-09-11

**Authors:** Qien Zhong, Jianghao Hu, Sheraz Ahmad, Xiaole Xie, Kang Li, Huiyu Yi, Ling Tian

**Affiliations:** 1 Guangdong Provincial Key Lab of Agro-Animal Genomics and Molecular Breeding, College of Animal Science, South China Agricultural University, Guangzhou 510642, China; 20231026004@stu.scau.edu.cn (Q.Z.); dh20010@yzu.edu.cn (S.A.);; 2 Guangxi Key Laboratory of Sericulture Ecology and Applied Intelligent Technology/Guangxi Collaborative Innovation Center of Modern Sericulture and Silk, Hechi University, Hechi 546300, China; 3 College of Agriculture, Guangxi University, Nanning 530004, China; 4 Sericulture Institute, Guangxi University, Nanning 530004, China

**Keywords:** autophagy, *Bombyx mori*, BmAtg8–PE formation, untargeted metabolomics, lipid metabolic reprogramming

## Abstract

Tissue-specific autophagic remodeling drives larval tissue dismantling during lepidopteran metamorphosis, yet the metabolic regulators underlying tissue-autonomous autophagic heterogeneity remain incompletely defined. This study sought to dissect the linkage between lipid metabolic reprogramming and tissue-divergent autophagic potency in *Bombyx mori*. Spatiotemporal autophagic levels across larval tissues was profiled via BmAtg8–PE immunoblotting, BmAtg8 immunofluorescence, and LysoTracker Red lysosomal staining. Untargeted metabolomics was conducted on the fat body, midgut, posterior silk gland, and trachea at key metamorphic stages; differentially accumulated metabolites (DAMs) were processed through hierarchical clustering and KEGG functional enrichment. Autophagic activity was universally upregulated upon prepupal transition, with the posterior silk gland and trachea showing markedly higher autophagic levels, whereas the fat body and midgut maintained relatively lower autophagic activity in tissue-wide comparison. Lipids represented the most dynamically remodeled metabolite families; fatty acyls and their synthetic cascades were enriched in high-autophagy tissues, while glycerolipid and glycerophospholipid pathways prevailed in low-autophagy tissues. Integrative multi-omics and cytological evidence identify a correlative relationship between tissue-resolved lipid turnover and spatially segregated autophagic activation. This tissue-scale atlas establishes a fundamental resource for mechanistic exploration of lipid-autophagy crosstalk governing insect metamorphic development.

## Simple Summary

Tissue-specific autophagy underpins tissue remodeling during lepidopteran metamorphosis, yet the metabolic determinants governing tissue-specific autophagic divergence remain largely uncharacterized. This study combined autophagic phenotypic assays and untargeted metabolomics to decipher lipid-autophagy coordination in *Bombyx mori*. Distinct autophagic stratification was identified across larval tissues: the posterior silk gland and trachea sustained high autophagic levels, whereas fat body and midgut exhibited muted autophagy. Lipids dominated the dynamically regulated metabolome, and clustering and KEGG enrichment revealed conserved lipid-subclass partitioning. High-autophagy tissues accumulated fatty acyls alongside activated fatty acid biosynthesis and phagolysosome pathways, while glycerolipid and glycerophospholipid metabolism prevailed in low-autophagy tissues. Our integrative data establishes a correlative link between tissue-resolved lipid reprogramming and spatially divergent autophagic potency, providing a tissue-level resource for exploring lipid-autophagy regulatory crosstalk in insect metamorphosis.

## 1. Introduction

Autophagy is an evolutionarily conserved homeostatic cascade that eliminates damaged organelles and misfolded proteins to sustain cellular quality control and metabolic balance [1,2]. In insects, this lysosome-dependent catabolic process governs multiple physiological events, including nutrient stress adaptation, immune defense, and metamorphic tissue remodeling, with tissue-specific autophagic responsiveness tailored to distinct tissue functional demands [3]. Larval–pupal metamorphosis triggers robust autophagic activation to clear obsolete larval tissues such as fat body, midgut, and silk glands; genetic ablation of autophagy impairs pupal epithelial differentiation and tissue degradation in *Bombyx mori* [4,5]. Parallel investigations in *Drosophila melanogaster* confirm that autophagy collaborates with programmed cell death to drive breakdown of midgut, intersegmental muscles, and fat body, and tissue-autonomous autophagy-dependent tissue dismantling has been widely documented across lepidopteran species [3].

Autophagosome biogenesis relies on a cohort of autophagy-related (Atg) proteins, among which Atg8 lipidation serves as a rate-limiting molecular checkpoint for autophagic flux [6,7]. Via covalent conjugation with phosphatidylethanolamine (PE), Atg8 forms the membrane-anchored Atg8–PE complex to facilitate phagophore expansion, membrane curvature, and autophagosome closure [8,9,10]. This lipidated modification is essential for cargo recruitment and autophagosome–lysosome fusion, and serves as a canonical biomarker for autophagic flux under developmental and stress stimuli [11]. Conserved signaling axes tightly control autophagic initiation: mechanistic target of rapamycin (mTOR) signaling suppresses autophagy under nutrient-replete conditions through phosphorylation of the ULK1/Atg1 complex [12,13], whereas the metamorphosis steroid hormone 20-hydroxyecdysone (20E) acts as a master inducer of autophagy during lepidopteran developmental transitions [14,15]. In *B. mori*, 20E elevates the transcription of core *Atg* genes and accelerates Atg8–PE conjugation to facilitate larval tissue clearance prior to pupation [16,17,18]. Mechanistically, Atg8 lipidation is catalyzed by two ubiquitin-like cascades mediated by Atg7 and Atg3, anchoring Atg8 onto nascent autophagic membranes to promote autophagosome-lysosome fusion and substrate catabolism [19,20,21,22,23,24,25].

As a classic lepidopteran model organism, *B. mori* has been extensively adopted to decipher autophagy-mediated developmental plasticity, nutrient redistribution, and oxidative stress resistance. During larval–pupal transition, widespread autophagy in fat body and midgut recycles intracellular macromolecules to support pupal morphogenesis [26,27], while tissue-specific autophagic activation mitigates oxidative damage to maintain organismal homeostasis [28,29,30]. Existing insect autophagy research predominantly focuses on upstream hormonal signaling and autophagosome assembly machinery, yet two fundamental knowledge gaps remain unresolved. First, systematic spatiotemporal profiling of graded autophagic flux across multiple functionally divergent larval tissues is lacking, and tissue-stratified autophagic signatures during silkworm metamorphosis have not been quantitatively characterized. Second, the metabolic landscape coupled to tissue-specific autophagic potency remains largely uncharted. Although lipids constitute essential structural and signaling substrates for autophagosome formation, it remains elusive whether conserved lipid subclass and pathway partitioning exists between high- and low-autophagy tissues, and how tissue-resolved lipid metabolic reprogramming coordinates spatially segregated autophagic activation.

To address these questions, the present study integrated multi-modal cytological autophagy detection and untargeted metabolomics to dissect lipid-autophagy coordination across representative silkworm tissues at key metamorphic stages. BmAtg8–PE immunoblotting, BmAtg8 immunofluorescence, and LysoTracker Red lysosomal acidification staining were jointly applied to map tissue- and stage-specific autophagic levels. Global metabolome profiling was subsequently performed on the fat body, midgut, posterior silk gland, and trachea, with differentially accumulated metabolites subjected to hierarchical clustering and Kyoto Encyclopedia of Genes and Genomes (KEGG) functional enrichment analyses. Our multi-layered phenotypic and metabolomic dataset delineates stable lipid metabolic divergence between low-autophagy tissues (fat body, midgut) and high-autophagy tissues (posterior silk gland, trachea), establishing a correlative linkage between tissue-specific lipid rewiring and differential autophagic intensity. This work constructs a tissue-level integrated atlas coupling autophagic dynamics and lipid metabolism, providing a foundational resource for further mechanistic exploration of lipid-autophagy crosstalk governing insect metamorphic development.

## 2. Materials and Methods

### 2.1. Insect Rearing and Tissue Collection

The *B. mori* (Dazao) larvae were reared on fresh mulberry leaves at 25 °C under a 14 h light/10 h dark cycle. Tissues, including the fat body, posterior silk gland, midgut, Malpighian tubules, trachea, ovary, and testis, were dissected from larvae at specific developmental stages (e.g., the 5th instar, wandering, and prepupal stages) and immediately frozen in liquid nitrogen for further analysis or subjected to immunofluorescent or LysoTracker staining following the procedure in previous studies [17,31].

### 2.2. Western Blot Analysis

Proteins were extracted from tissue samples using RIPA lysis buffer (Beyotime Biotechnology, Shanghai, China, P0013B) containing a protease inhibitor cocktail (Merck KGaA, Darmstadt, Germany, 11836170001). The extracted proteins were separated by SDS-PAGE and subsequently transferred to PVDF membranes using standard immunoblotting procedures as previously described [4]. Primary antibodies were applied at the following dilutions: anti-BmAtg8–PE (Abcam, Cambridge, MA, USA, ab109364; 1:4000, *v*/*v*) and anti-tubulin (tubulin alpha 1a, Beyotime Biotechnology, AT819; 1:5000, *v*/*v*)), both used according to the manufacturers’ recommendations. Protein bands were detected using an appropriate HRP-conjugated secondary antibody and visualized with an enhanced chemiluminescence (ECL) system (Yeasen Biotechnology Co., Ltd., Shanghai, China). Band intensities were quantified using ImageJ software (version 1.46; National Institutes of Health, Bethesda, MD, USA; http://rsbweb.nih.gov/ij (accessed on 1 September 2025)). Three biological replicates were performed. For each replicate, tissues were collected from 10 individuals and pooled.

### 2.3. Immunofluorescent Staining

Tissues were collected from larvae at specific developmental stages and then fixed in 4% paraformaldehyde in 0.1 M PBS (pH 7.5) at 4 °C for 6 h, followed by additional fixation in 4% paraformaldehyde (in PBS, pH 10.4) at 4 °C overnight. Immunofluorescent staining was performed using a standard protocol as previously described [32]. Samples were incubated overnight at 4 °C with a primary antibody against BmAtg8 (1:100, *v*/*v*), followed by a 2 h incubation at room temperature with a fluorescent secondary antibody conjugated to Alexa Fluor 488 (Abcam, Cambridge, MA, USA, ab150077; 1:200, *v*/*v*). Immunofluorescent signals were observed under a confocal microscope (Olympus FV3000, Olympus Corporation, Tokyo, Japan).

### 2.4. LysoTracker Red Staining

Newly collected tissues from larvae at different developmental stages, including perivisceral fat body (from the 5th abdominal segment), posterior silk gland, midgut, Malpighian tubules, trachea, ovary, and testis, were separated into small pieces by forceps and thoroughly washed with PBS (pH 7.0, 0.1 M). The newly collected tissues were stained with LysoTracker Red DND-99 (Invitrogen, Thermo Fisher Scientific, Waltham, MA, USA, L7528) following a previously described protocol [4]. LysoTracker Red staining was observed under the confocal microscope ((Olympus FV3000, Olympus Corporation, Tokyo, Japan).

### 2.5. Sample Processing and LC/MS/MS Analysis for the Metabolites

Frozen silkworm tissue specimens stored at −80 °C were processed with rigorous pre-treatment procedures to remove impurities and preserve endogenous small-molecule metabolites. Briefly, tissues were homogenized, spiked with internal standards, vortexed, subjected to ice-bath ultrasonic extraction, and centrifuged sequentially at 14,000× *g* for 20 min at 4 °C for crude metabolite collection. The resulting supernatants were lyophilized, oximated, and chemically derivatized to enhance chromatographic retention and ionization efficiency of polar metabolites prior to instrumental detection. All metabolomic profiling datasets were obtained from six independent biological replicates per group. Untargeted metabolomic profiling was performed on a reversed-phase liquid chromatography-tandem mass spectrometry (RPLC-MS/MS) platform combining an Agilent 1290 Infinity UHPLC system (Agilent Technologies, Santa Clara, CA, USA) and an AB Sciex TripleTOF 6600 quadrupole time-of-flight mass spectrometer (Sciex, Framingham, MA, USA).

Metabolite separation was conducted using a Waters ACQUITY UPLC HSS-T3 column (2.1 mm × 100 mm, 1.8 μm). Distinct mobile phase formulations were used for electrospray ionization positive (ESI+) and negative (ESI−) modes. For ESI+ mode, the mobile phases consisted of ultrapure water with 0.1% formic acid (phase A) and acetonitrile with 0.1% formic acid (phase B). For ESI− mode, 0.5 mM aqueous ammonium fluoride and pure acetonitrile served as phase A and phase B, respectively. The elution gradient was initialized with 1% phase B for 1.5 min, linearly increased to 99% phase B over 11.5 min, and held for 3.5 min to elute retained metabolites. The column was then rapidly reset to 1% phase B within 0.1 min and re-equilibrated for 3.4 min to guarantee stable chromatographic conditions for subsequent sample injection. All chromatographic runs were performed at a flow rate of 0.3 mL/min and a column temperature of 25 °C, with a sample injection volume of 2 μL.

All mass spectrometric analyses were acquired with optimized ESI source parameters. Ion source gas 1 and gas 2 were set to 40 psi and 80 psi, respectively, with the curtain gas maintained at 30 psi. The ion source temperature was fixed at 600 °C, and the ion spray floating voltage was set to 5000 V for ESI+ mode and −4000 V for ESI− mode. Full-scan TOF-MS spectra were captured across an m/z range of 60–1000 Da, with a scanning accumulation time of 0.05 s per spectrum. Information-dependent acquisition (IDA) was operated in high-sensitivity mode to trigger automatic secondary fragment ion scanning. MS2 fragmentation was performed at a collision energy of 35 ± 15 eV, and the declustering potential was set to ±60 V to maximize ion transmission and metabolite fragment identification accuracy.

### 2.6. Metabolite Data Processing

The raw data (wiff.scan files) were converted to MzXML format using ProteoWizard MSConvert, and the resulting files were analyzed with XCMS for feature detection. Retention time correlation and alignment. All metabolomic features acquired from both positive and negative electrospray ionization modes were merged and redundantly filtered prior to all subsequent integrated bioinformatic analyses. The metabolites were identified with high precision mass accuracy (<25 ppm) and MS/MS/data, which were compared to our standards database. Only variables with more than 50% nonzero measurement values in at least one group were retained in the retrieved ion features. The MetaboAnalyst (www.metaboanalyst.ca, accessed on 12 September 2025) web-based platform was used for multivariate statistical analysis. After Pareto scaling, principal component analysis (PCA) and partial least squares discrimination analysis (PLS-DA) were conducted. Leave-one-out cross-validation and response permutation testing were used to assess the model’s robustness. Significant metabolites were identified by combining three criteria: a statistically significant threshold of variable importance in projection (VIP) values from the PLS-DA model, two-tailed Student’s *t*-test results for raw data, and fold change (FC) values. Specifically, metabolites with a variable importance in projection (VIP) > 1, *p*-value < 0.05, and |log_2_fold change (FC)| ≥ 1 were considered differentially abundant metabolites.

### 2.7. KEGG Analysis of Differential Metabolites

For functional annotation and pathway profiling of differential metabolites, corresponding Kyoto Encyclopedia of Genes and Genomes (KEGG) compound IDs were converted and retrieved via the ID-matching module of the MBRole online platform (http://csbg.cnb.csic.es/mbrole/, accessed on 15 September 2025) [33]. Validated KEGG identifiers were subsequently adopted for metabolic pathway enrichment analysis to systematically characterize altered metabolic patterns across grouped samples [34,35]. This workflow further facilitated the integrated annotation of enriched metabolic pathways and their corresponding catalytic enzymes. Statistical significance was defined by a threshold of *p* < 0.05, where lower *p*-values indicated more robust and reliable pathway enrichment differences between comparative groups.

### 2.8. Statistical Analysis

Experimental data were analyzed in GraphPad Prism 10 software version 10.5.0 (774); differences between groups were analyzed using an unpaired two-tailed Student’s *t*-test, and two-way ANOVA with Tukey’s multiple comparisons test was applied for comparisons between more than two groups.

## 3. Results

### 3.1. Expression Profiles of BmAtg8–PE Protein in Different Tissues and Developmental Stages of B. mori

**Figure 1:**
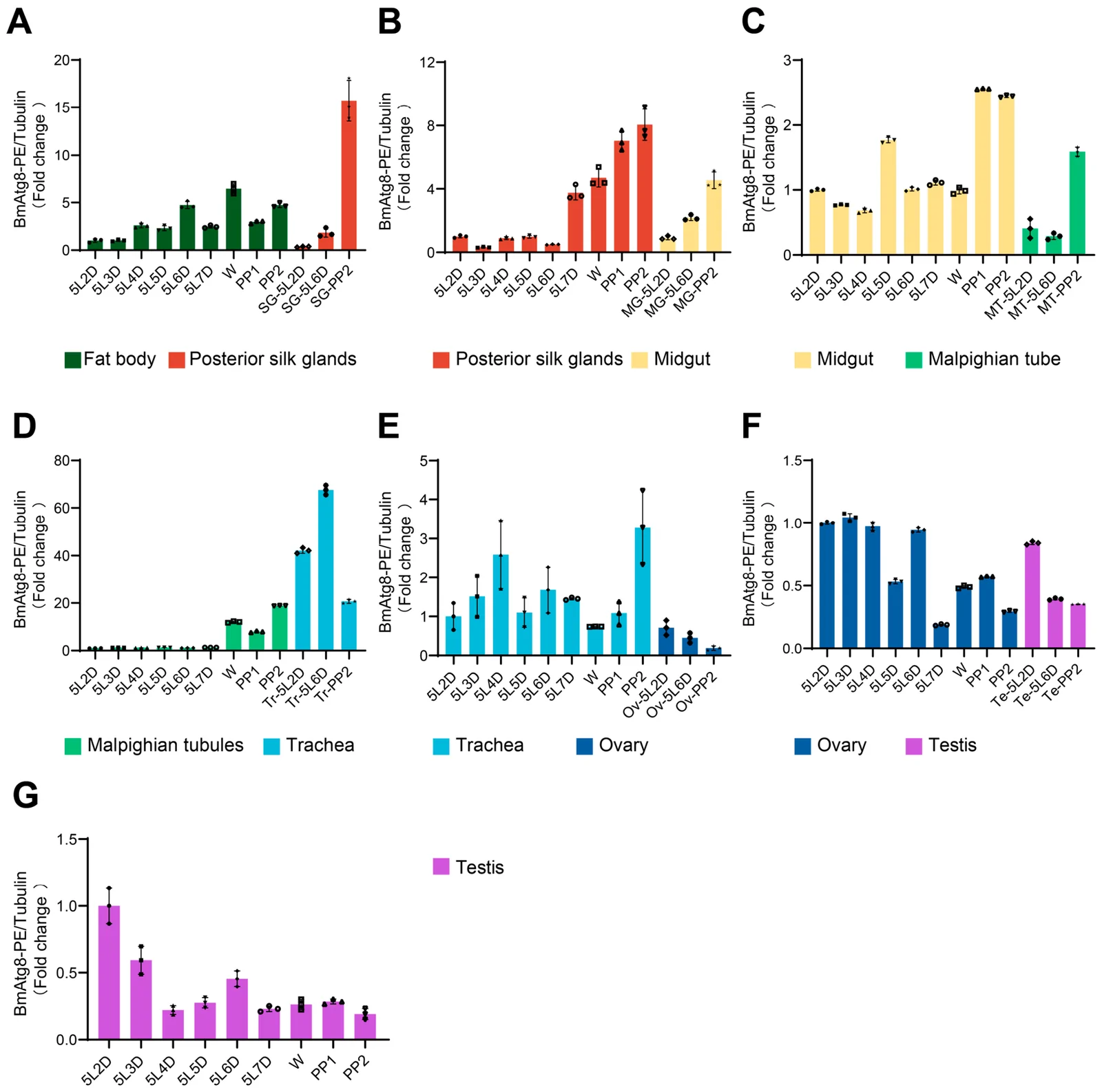
Gray value quantification of BmAtg8–PE bands of different tissues in *B. mori*. (**A**) represents the fat body and posterior silk gland, (**B**) shows the posterior silk gland and midgut, (**C**) displays the midgut and Malpighian tubules, (**D**) presents the Malpighian tubules and trachea, (**E**) depicts the trachea and ovary, (**F**) highlights the ovary and testis, and (**G**) examines the testis. Relative BmAtg8–PE levels were normalized to the 5L2D group of each tissue, with fold changes presented for all tested stages and tissues (*n* = 3).

Western blot analysis revealed that BmAtg8–PE was differentially expressed in various tissues of *B. mori* across day 2 of the 5th instar (5L2D) to day 2 of prepupa (PP2) (Figure 1 and Figure S1). In most tissues of the late-stage silkworm larvae (e.g., fat body, posterior silk gland, midgut, Malpighian tubules, and trachea), the level of BmAtg8–PE was maintained at a high abundance, which was particularly pronounced during the larval–pupal transition. Notably, the BmAtg8–PE level peaked at the PP2 stage in the posterior silk gland and trachea (Figure 1B,E and Figure S1B,E). Intriguingly, an entirely opposite expression pattern was observed in ovary and testis tissues. Both testis and ovary exhibited relatively high BmAtg8–PE levels at the early 5th instar stage (Figure 1E–G and Figure S1E–G). Autophagic levels was evaluated based on the relative abundance of the lipidated BmAtg8–PE form, the canonical marker of autophagosome formation. Overall, pairwise comparisons of BmAtg8–PE accumulation in various tissues at the larval–pupal transition stage showed that this lipidated protein was present at the highest levels in the posterior silk gland and trachea, comparatively low levels in the midgut and fat body, and moderate, steady levels in gonads.

The analysis includes multiple tissue types, where panel (A) represents the fat body and posterior silk gland, (B) the posterior silk gland and midgut, (C) midgut and Malpighian tubules, (D) Malpighian tubules and trachea, (E) trachea and ovary, (F) ovary and testis, and (G) the testis. The developmental stages assessed encompass 5L2D, 5L3D, 5L4D, 5L5D, 5L6D, 5L7D, the wandering stage (W), day 1 of prepupa (PP1), and day 2 of the prepupa (PP2). M: molecular weight markers.

### 3.2. Immunofluorescent Staining of BmAtg8 in Various Tissues of B. mori

**Figure 2:**
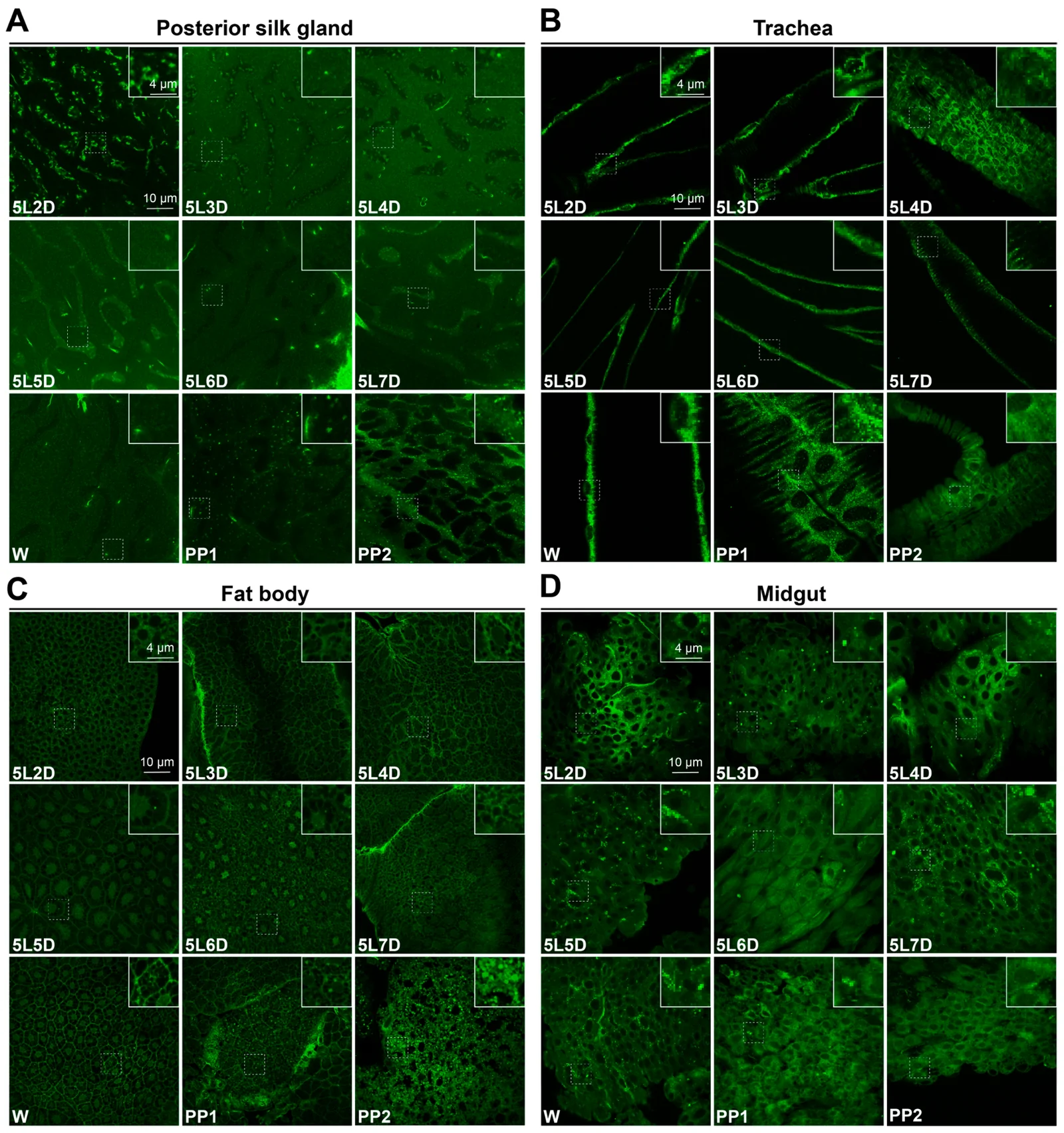
Immunofluorescent staining of various tissues across different developmental stages. Representative immunofluorescence micrographs display the expression and subcellular distribution of BmAtg8 across key developmental stages. The developmental stages assessed encompass 5L2D-5L7D, the wandering stage (W), day 1 of the prepupa (PP1), and day 2 of the prepupa (PP2). Panels show distinct larval tissues: (**A**) posterior silk gland; (**B**) trachea; (**C**) fat body; (**D**) midgut. Green punctate signals represent BmAtg8 protein aggregates. White square boxes indicate regions magnified in the insets. Immunofluorescence images for the remaining three tissues (Malpighian tubules, ovary, and testis) are provided in Supplementary Figure S2.

To elucidate the variations in autophagy throughout development, we analyzed the punctation of BmAtg8 across multiple tissues of *B. mori* using immunofluorescent staining. Seven tissues in total were subjected to immunofluorescence analysis. To facilitate subsequent integrated comparison with our metabolomic dataset, four tissues covered by both analyses are presented in the main figure (Figure 2), while the remaining three tissues (Malpighian tubules, ovary, and testis) are shown in Figure S2. The immunofluorescence observation of BmAtg8 revealed distinct spatiotemporal patterns across tissues and developmental stages (Figure 2). In silk glands and midgut, intense BmAtg8 fluorescence persisted from the 5th instar stage feeding period through the wandering and prepupal stages. By contrast, weak BmAtg8 signals were detected in trachea and fat body at early fifth instar, with obvious elevation during larval–pupal transition (Figure 2). Malpighian tubules only displayed measurable BmAtg8 fluorescence at larval–pupal metamorphosis (Figure S2A). In ovary and testis tissues, fluctuating levels of BmAtg8 fluorescence were observed throughout the 5th instar, with a pronounced increase during the larval-pupal metamorphosis (Figure S2B,C). In summary, tissues including the fat body and Malpighian tubules exhibited few BmAtg8 puncta during the fifth-instar feeding stage, yet abundant BmAtg8 punctate signals were detected in all tissues at larval–pupal transition. This dynamic distribution of BmAtg8 puncta matched the accumulation pattern of BmAtg8–PE, indicating synchronized autophagic activation during metamorphosis.

### 3.3. LysoTracker Red Staining in Various Tissues of B. mori

**Figure 3:**
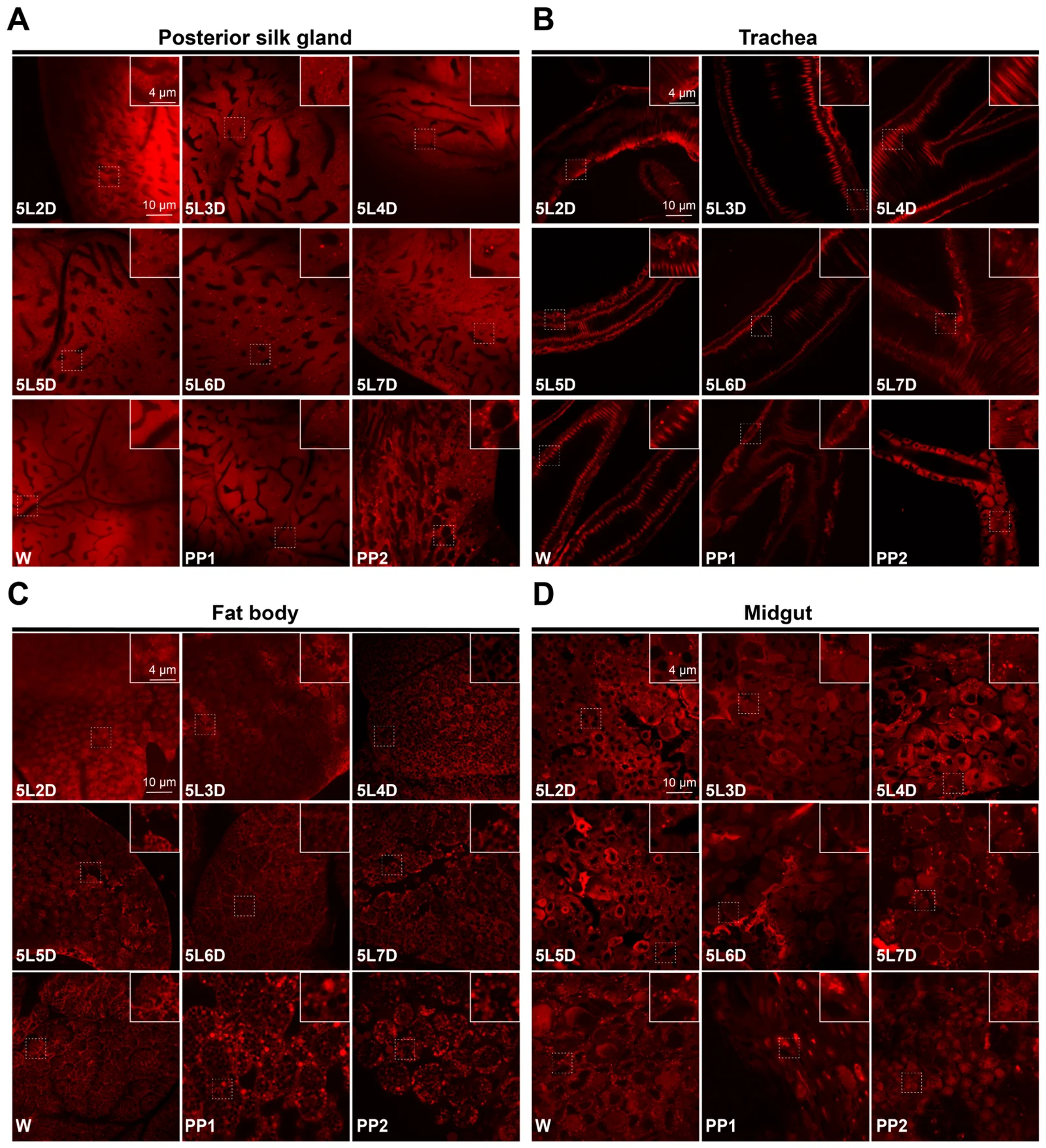
LysoTracker Red staining of various tissues across different developmental stages.

Tissue and stage-specific variations in BmAtg8–PE levels in *B. mori* suggest that autophagy is spatiotemporally regulated during development. To further investigate autophagic levels, we performed LysoTracker Red staining across multiple tissues and developmental stages. Seven tissues in total were subjected to LysoTracker staining. To enable subsequent cross-comparison with metabolomic data, four tissues common to both analyses are displayed in the main figure (Figure 3), whereas the remaining three tissues (Malpighian tubules, ovary, and testis) are provided in Figure S3. Silk glands and midgut exhibited highly similar lysosomal acidification dynamics. More specifically, strong lysosomal acidification was observed in silk glands before the wandering stage, matching the high BmAtg8–PE abundance within this tissue (Figure 3A). In parallel, the midgut maintained strong acidification from 5L2D through the wandering stage (Figure 3D). On the contrary, the trachea and fat body only showed weak acidification signals at the early 5th instar stage, whereas a dramatic increase occurred during larval–pupal transition (Figure 3B,C). Similarly, Malpighian tubules produced measurable acidification solely at metamorphosis (Figure S3A). As for the ovary and testis, their lysosomal acidification remained generally stable with minor fluctuations throughout the 5th instar stage (Figure S3B,C). Taken together, the overall variation trend of lysosomal acidification in diverse tissues was consistent with the elevated accumulation of BmAtg8–PE. In summary, all examined tissues displayed enhanced lysosomal acidification during the larval-pupal transition, indicating a coordinated activation of this process to support metamorphosis.

The developmental stages assessed including 5L2D-5L7D, the wandering stage (W), day 1 of the prepupa (PP1), and day 2 of the prepupa (PP2). Panels show distinct larval tissues: (A) posterior silk gland; (B) trachea; (C) fat body; (D) midgut. White square boxes indicate regions magnified in the insets. LysoTracker staining results for the remaining three tissues (Malpighian tubules, ovary, and testis) are provided in Supplementary Figure S3.

### 3.4. Metabolite Profiles of Different Tissues in B. mori

**Figure 4:**
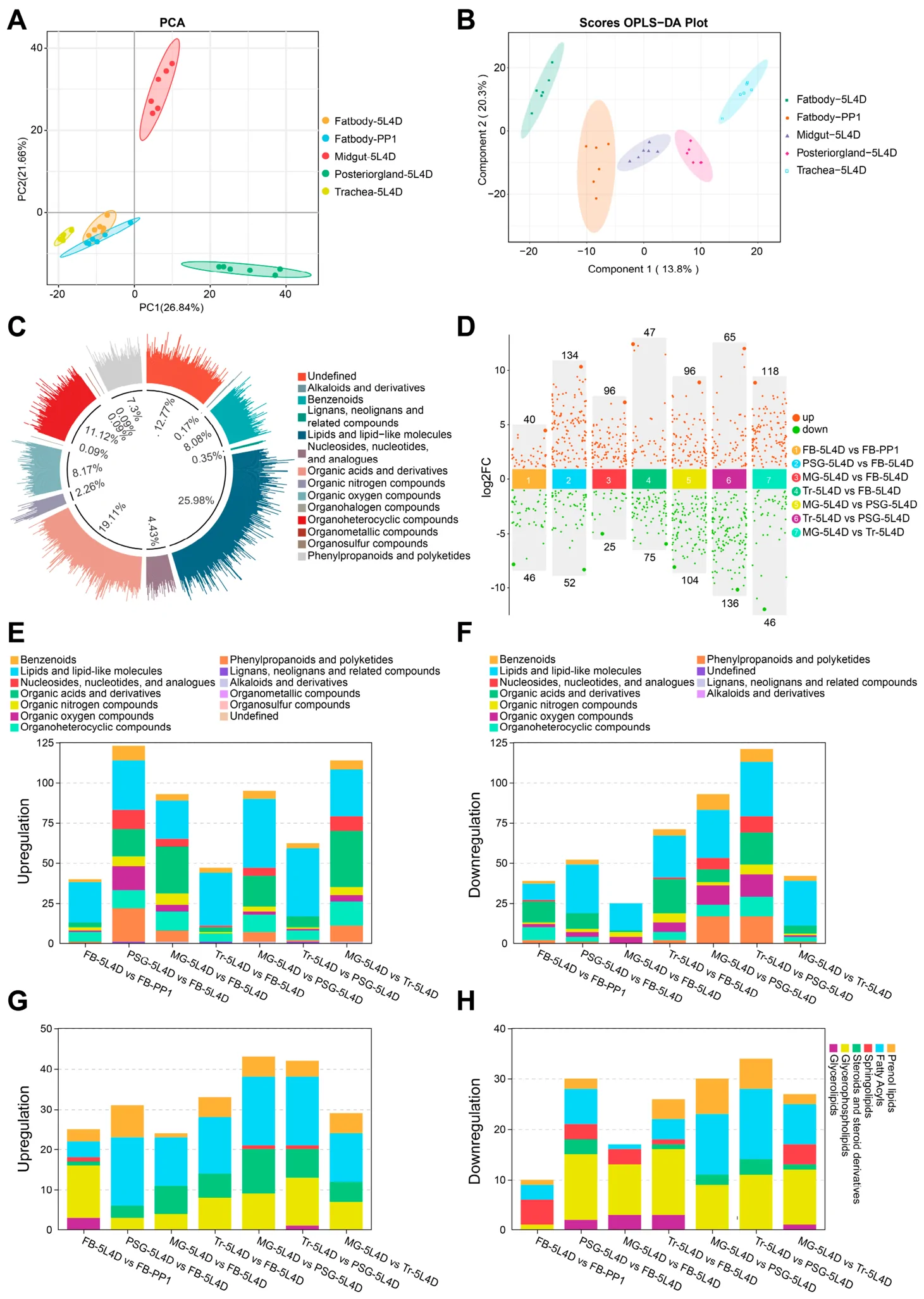
Global untargeted metabolomics reveals tissue-specific metabolic signatures in silkworm larval tissues during metamorphosis. (**A**) PCA score plot: PC1 (26.84%) and PC2 (21.66%) separate FB-5L4D, FB-PP1, MG-5L4D, PSG-5L4D and Tr-5L4D; ellipses represent 95% confidence intervals. (**B**) OPLS-DA score plot with Component 1 (13.8%) and Component 2 (20.3%), showing distinct metabolic clustering of sample groups. (**C**) Circular plot displaying proportional distribution of annotated metabolites across chemical superclasses. (**D**) Volcano plots of DAMs for seven pairwise comparisons. X-axis: log_2_FC; orange/green dots represent upregulated/downregulated metabolites, with DAMs counts labeled. Abbreviations: FB, fat body; MG, midgut; PSG, posterior silk gland; Tr, trachea. All abbreviations apply to subsequent figures and tables. (**E**,**F**) Stacked bar charts of upregulated (**E**) and downregulated (**F**) DAM counts classified by metabolite superclass. (**G**,**H**) Stacked bar charts showing lipid subclass composition of upregulated (**G**) and downregulated (**H**) DAMs across all comparisons.

To resolve tissue- and stage-dependent metabolic changes associated with autophagy, we conducted untargeted metabolomics profiling of fat body (FB, 5L4D and PP1), midgut (MG), posterior silk gland (PSG), and trachea (Tr) at 5L4D. PCA and OPLS-DA (Figure 4A,B) revealed prominent separation of metabolic clusters across groups. The posterior silk gland and trachea with high autophagic activity exhibited unique metabolic features relative to the low-autophagy midgut and fat body, and fat body from 5L4D and PP1 displayed greatly divergent metabolic profiles, consistent with metamorphosis-induced autophagy activation in fat body. In total, 1152 metabolites were annotated from all samples (Table S1). Lipids and lipid-like molecules, along with organic acids and derivatives, represented the two most abundant metabolite categories, accounting for 25.98% and 19.11% of the total pool, respectively (Figure 4C). Differentially accumulated metabolites (DAMs) were screened using the thresholds of VIP > 1, |log2FC| > 1, and *p*-value < 0.05. Volcano plot analysis identified only 40 upregulated and 46 downregulated DAMs in the fat body at 5L4D versus PP1, indicating relatively moderate metabolic variation during the larval–pupal transition. By comparison, tissues collected at the 5L4D stage showed extensive metabolic rewiring compared with fat body. Specifically, the posterior silk gland contained 134 upregulated and 52 downregulated DAMs, and the midgut had 96 upregulated and 25 downregulated DAMs (Figure 4D). Consistent with the differential autophagic characteristics observed above, tissues with stronger autophagic activity harbored more upregulated DAMs than the fat body with weak autophagy, implying that intense autophagy may be closely associated with extensive tissue metabolic remodeling.

We next classified and quantified upregulated and downregulated DAMs based on chemical classification across all comparison groups (Figure 4E,F). Lipids and lipid-like molecules dominated both up- and downregulated DAMs, followed by organic acids and derivatives. Further lipid subclass analysis revealed that glycerophospholipids, sphingolipids, and fatty acyls were the most markedly altered lipid subsets (Figure 4G,H). Notably, the PP1 fat body with elevated autophagy was enriched in sphingolipids (e.g., sphingomyelin (Sm d30:1), N-lauroyl-d-erythro-sphingosylphosphorylcholine) and fatty acyls (e.g., acetylcarnitine, 5-aminovaleric acid betaine), whereas glycerophospholipids (e.g., LPC 18:1, β-glycerophosphate) predominated in the low-autophagy fat body at the 5L4D feeding stage. A comparable pattern was observed among 5L4D tissues: glycerophospholipids were more abundant in the fat body, while fatty acyls were highly accumulated in the posterior silk gland, midgut, and trachea—tissues with stronger autophagic activity. Overall, these distinct lipid subtype distributions correlate closely with autophagic intensity, suggesting that this distinct lipid reprogramming coincides with tissue-specific autophagic profiles during larval–pupal metamorphosis.

### 3.5. Clustering and Venn Analyses of Differentially Accumulated Metabolites Reveal Tissue-Specific Metabolic Distribution Patterns

**Figure 5:**
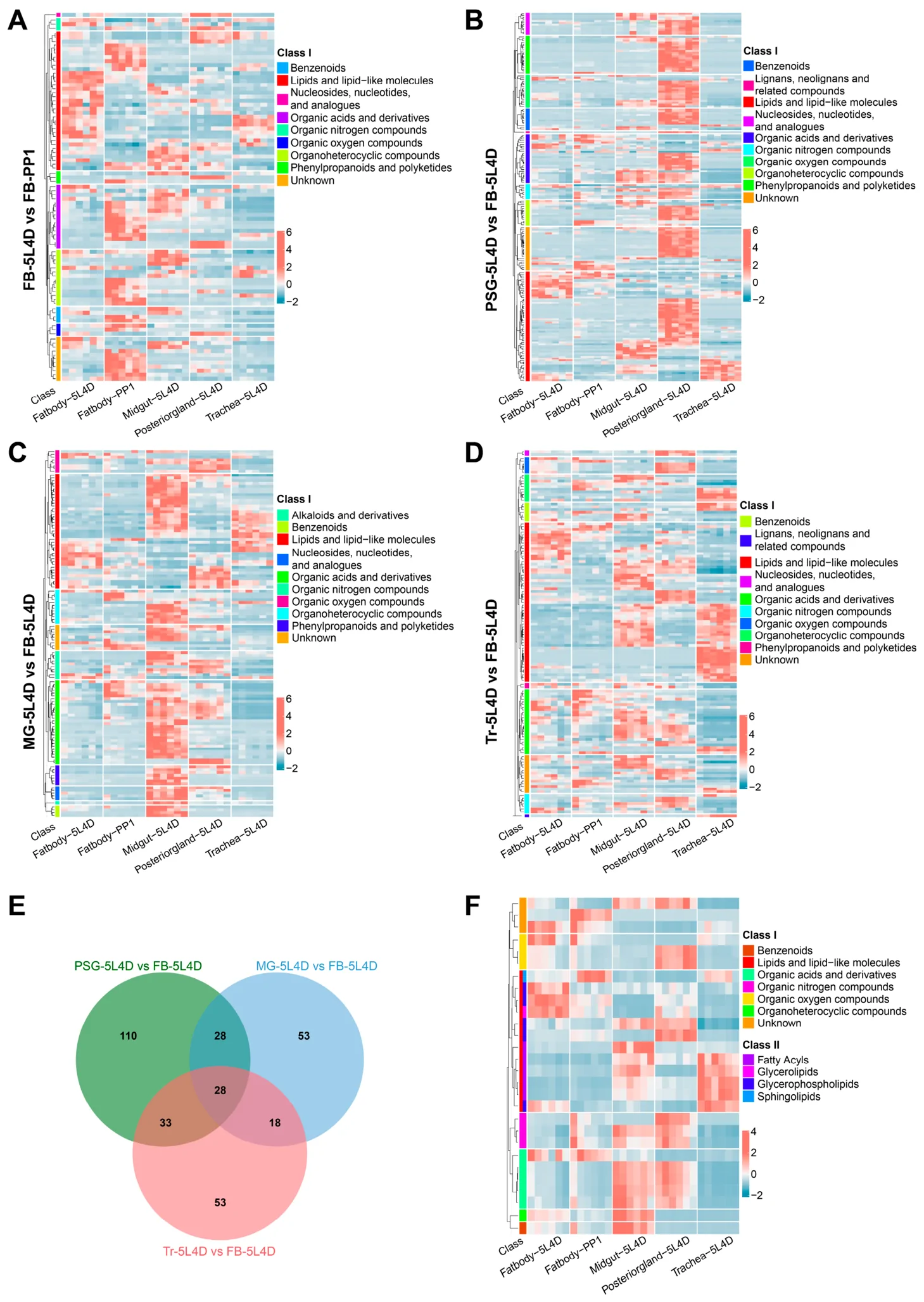
Tissue-specific metabolite clustering and overlapping differentially accumulated metabolites (DAMs) across *B. mori* larval tissues. (**A**–**D**) Heatmaps of DAMs from four pairwise comparisons: (**A**) FB-5L4D vs. FB-PP1; (**B**) PSG-5L4D vs. FB-5L4D; (**C**) MG-5L4D vs. FB-5L4D; (**D**) Tr-5L4D vs. FB-5L4D. Rows are annotated to metabolite superclasses (left color bars); columns represent tested tissue samples. Color gradient denotes relative metabolite abundance (red = high, blue = low). (**E**) Venn diagram illustrating unique and shared DAMs from PSG-5L4D vs. FB-5L4D, MG-5L4D vs. FB-5L4D, and Tr-5L4D vs. FB-5L4D, with numbers indicating metabolite counts. (**F**) Heatmap of the 28 core shared DAMs from pairwise comparisons of posterior silk gland, midgut, trachea versus fat body. Rows are grouped into lipid subclasses as indicated by the left color bar; the color scale reflects relative lipid abundance across all tissues.

Hierarchical clustering heatmaps categorized by chemical classification were generated for each pairwise comparison group to visualize the abundance distributions of differentially accumulated metabolites (DAMs). Marked fluctuations in lipid and lipid-like molecule abundance were observed across all comparisons, spanning tissues with divergent autophagic activity and distinct developmental stages. For most other metabolite superclasses, higher abundance was consistently detected in tissues with elevated autophagic activity. Notably, this trend was not fully conserved in trachea-involved comparisons, where only lipids and lipid-like molecules still exhibited prominent abundance variations (Figure 5A–D and Figure S4).

Venn diagram analysis further identified 28 shared DAMs across the three pairwise comparisons of posterior silk gland, midgut, trachea versus fat body at the 5L4D stage (Figure 5E). The corresponding heatmap profiles of these shared DAMs recapitulated the aforementioned lipid distribution pattern: relative to fat body at 5L4D, tissues with stronger autophagic activity (posterior silk gland and trachea) accumulated higher levels of fatty acyls. In contrast, the fat body and midgut with weaker autophagy exhibited greater enrichment of glycerolipids and glycerophospholipids (Figure 5F). Collectively, these observations imply that tissue-specific shifts in lipid composition may be closely associated with differential autophagic activity during silkworm metamorphosis.

### 3.6. KEGG Enrichment Reveals Stage- and Tissue-Specific Lipid Pathway Remodeling

**Figure 6:**
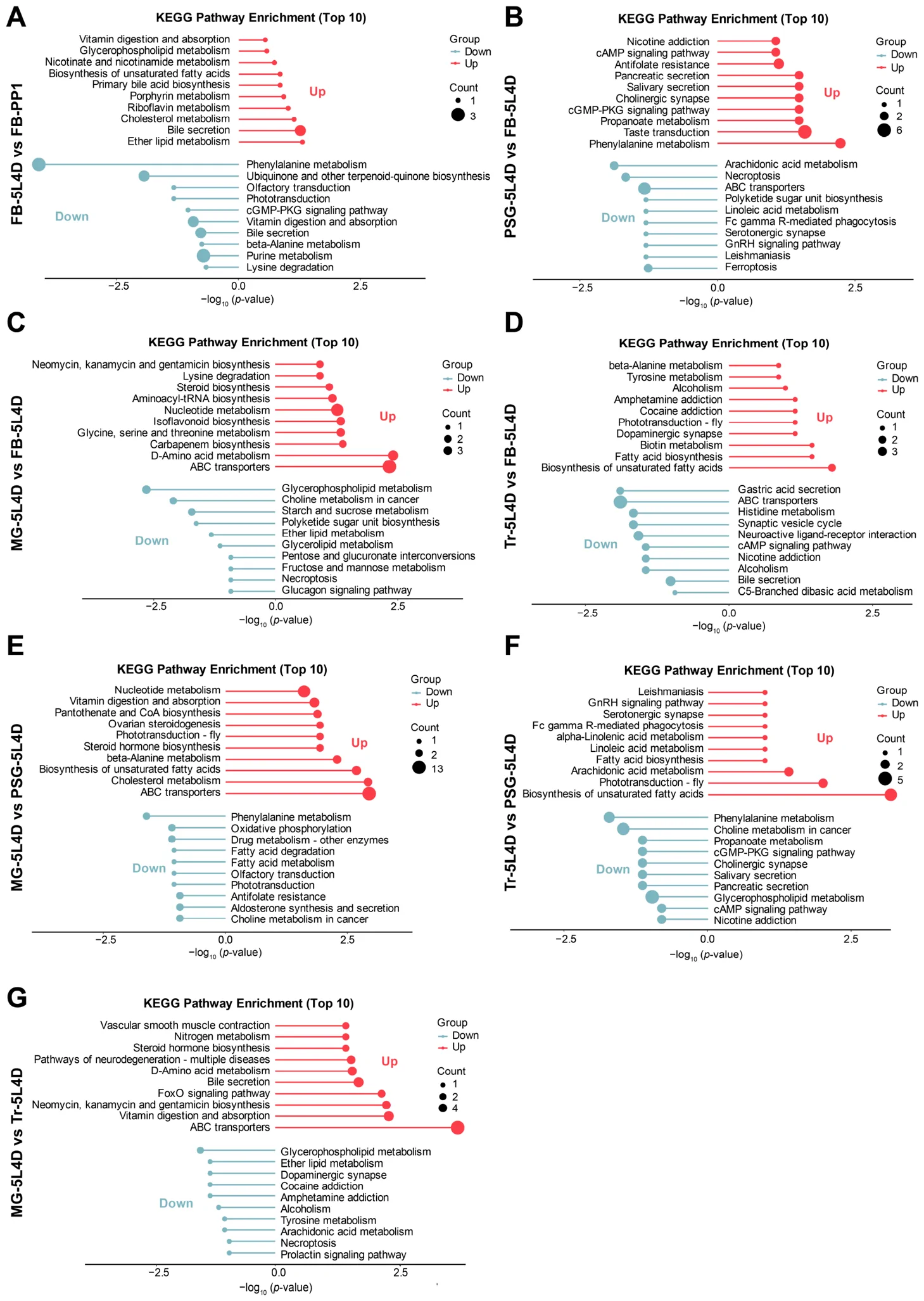
Top 10 enriched KEGG pathways of differentially accumulated metabolites (DAMs) across seven pairwise tissue comparisons. (**A**–**G**) Lollipop plots showing the top 10 significantly enriched KEGG pathways for each comparison: (**A**) FB-5L4D vs. FB-PP1; (**B**) PSG-5L4D vs. FB-5L4D; (**C**) MG-5L4D vs. FB-5L4D; (**D**) Tr-5L4D vs. FB-5L4D; (**E**) MG-5L4D vs. PSG-5L4D; (**F**) Tr-5L4D vs. PSG-5L4D; (**G**) MG-5L4D vs. Tr-5L4D. Red lollipops correspond to pathways enriched in upregulated DAMs, light blue lollipops represent pathways enriched in downregulated DAMs. Horizontal line length denotes −log_10_ (*p*-value), and dot size indicates the number of enriched DAMs in each pathway.

KEGG functional enrichment was performed on DAMs across all pairwise contrasts, with the top 10 pathways visualized based on −log_10_ (*p*-value) (Figure 6). Intra-fat body contrast (FB-5L4D vs. FB-PP1) exhibited prominent activation of lipid metabolic axes, including glycerophospholipid, cholesterol, and steroid-related biosynthesis (KEGG: primary bile acid biosynthesis), accompanied by repressed amino acid metabolism and cGMP-PKG signaling, highlighting lipid turnover as a core metabolic signature accompanying metamorphic metabolic shifts (Figure 6A). At the 5L4D feeding stage, comparisons of high-autophagy tissues (posterior silk gland and trachea) versus fat body revealed significant downregulation of arachidonic acid metabolism and the ABC transporter pathway, whereas fatty acid biosynthesis and phagolysosome-related pathways were enriched among upregulated DAMs; glycerophospholipid metabolism was universally suppressed in all tissue-versus-fat body comparisons (Figure 6B–D). In comparisons between midgut and posterior silk gland/trachea, the ABC transporter pathway was enriched in upregulated metabolites of midgut, alongside suppressed fatty acid biosynthesis and altered glycerolipid and glycerophospholipid metabolism (Figure 6E–G). Taken together, low-autophagy tissues (fat body, midgut) and high-autophagy tissues (posterior silk gland, trachea) exhibit consistent divergence in lipid species and enriched functional pathways. Metabolites belonging to the ABC transporter pathway tend to accumulate in low-autophagy tissues relative to high-autophagy counterparts. These findings recapitulate the tissue-resolved lipid distribution patterns above, implying that tissue-specific lipid reprogramming coupled with distinct ABC transporter-associated metabolic signatures may correlate with divergent autophagic potency across silkworm tissues during larval–pupal metamorphosis.

## 4. Discussion

Autophagy is a conserved lysosomal cascade driving larval tissue dismantling during lepidopteran metamorphosis, with organ-stratified autophagic flux tailored to distinct organ physiological demands [36]. The larval–pupal transition elevates transcription and lipidation of core *Atg* genes, including *BmAtg8*, generating tissue-specific autophagic profiles: the posterior silk gland and trachea exhibit robust autophagy, while the fat body and midgut show weak autophagic signals across the fifth instar [37,38,39,40,41]. Orthogonal cytological assays, including BmAtg8–PE immunoblotting, immunofluorescence, and LysoTracker staining, validated this spatial partitioning; cytoplasmic BmAtg8 puncta at metamorphic peaks reflect active autophagosome biogenesis, with Atg8–PE lipidation serving as a canonical marker for autophagic flux [11]. Despite well-characterized Atg8 lipidation machinery [19,20,21,22,23,24,25], the tissue-resolved lipid metabolic landscape coupled to graded tissue autophagy remains poorly characterized in silkworms.

Untargeted metabolomics uncovered lipids as the most dynamically modulated metabolites. Subsequent clustering and KEGG enrichment analyses revealed clear segregation of lipid subclasses between high- and low-autophagy tissues. Fatty acids and their biosynthetic pathways accumulated in the posterior silk gland and trachea, whereas glycerolipid and glycerophospholipid metabolism predominated in the fat body and midgut. Widespread mTOR pathway enrichment supports the established model that 20E suppresses mTORC1 to trigger systemic metamorphic autophagy [14,42], while tissue-specific carbon and glutathione metabolic shifts match organ-specific energy supply and oxidative stress tolerance requirements [43,44,45]. Cross-taxa studies have confirmed bidirectional coordination between lipid metabolism and autophagy: fatty acyls supply membrane substrates for phagophore expansion, whereas excess glycerophospholipids negatively modulate autophagic initiation via AMPK inhibition [13]. Consistent with this conserved regulatory circuit, metabolomic profiling of *Helicoverpa armigera* demonstrated stage-specific lipid redistribution to support nutrient recycling during metamorphic tissue remodeling [46].

As the primary insect metabolic tissue, the fat body exhibited the most prominent stage-dependent metabolomic shifts between 5L4D and PP1, consistent with our previous work establishing lipid-autophagy feedback regulation in the silkworm fat body. Autophagy deficiency leads to glycerophospholipid accumulation and subsequent autophagy suppression through impaired AMPK signaling [4]. Our multi-tissue lipid profiling extends this single-tissue model, revealing that graded partitioning of glycerolipids and fatty acyls correlates with divergent autophagic intensity across larval tissues. Parallel research in *Chilo suppressalis* further verified that 20E-mediated fatty acids metabolic reprogramming coordinates tissue-specific autophagy to facilitate pupal tissue development [47]. Integrative cytological and metabolomic data reveal a correlative association between tissue-specific lipid metabolic profiles and spatially segregated autophagic levels during silkworm metamorphosis.

Notably, KEGG enrichment identified specific upregulation of the ABC transporter pathway in low-autophagy tissues (fat body and midgut), representing a unique tissue-specific metabolic signature associated with lipid homeostasis and autophagic modulation. In *B. mori*, ABC transporters facilitate lipid trafficking and maintain cellular lipid metabolism [48]. This pathway enrichment implies a compensatory metabolic strategy: impaired autophagic lipid recycling in low-autophagy tissues is remedied by ABC transporter-dependent lipid transport, which prevents aberrant intracellular lipid accumulation. This lipid remodeling reshapes the substrate pool for BmAtg8–PE lipidation and autophagosome biogenesis, and this lipid distribution pattern coincides with tissue-specific autophagic profiles during metamorphosis.

This study carries inherent correlative limitations without tissue-specific functional perturbation. Subsequent tissue-restricted RNAi or CRISPR/Cas9 targeting core *BmAtg* and lipid metabolic enzymes, combined with paired metabolomic and autophagic flux quantification, will resolve the directional regulatory relationship between them [49]. Only four representative larval organs were analyzed here; expanded tissue coverage and targeted lipidomics of differential metabolites will further refine the lipid-autophagy regulatory framework.

In summary, this work constructs a multi-tissue dataset characterizing spatiotemporal autophagic dynamics and accompanying lipid metabolic changes during silkworm larval–pupal metamorphosis. Autophagic activity displays clear tissue-specific patterns matching organ functional demands, and stable divergence in lipid subclasses and metabolic pathways distinguishes tissues with contrasting autophagic levels. Our results revise the unidirectional view of autophagy-driven metabolic remodeling and demonstrate that lipid metabolic reprogramming coincides with graded autophagic activation across tissues. This tissue-level dataset supplements current understanding of developmental crosstalk between lipid metabolism and autophagy in lepidopterans, providing a foundational reference for further mechanistic exploration of metamorphic tissue remodeling.

## 5. Conclusions

This study systematically profiled spatiotemporal autophagic levels in multiple *Bombyx mori* tissues during larval–pupal metamorphosis via BmAtg8–PE immunoblotting, immunofluorescence, and LysoTracker staining. Autophagy was broadly induced at the prepupal stage with evident tissue heterogeneity in autophagic activity: the posterior silk gland and trachea showed high autophagic activity and the fat body and midgut exhibited weak autophagy, while the gonads maintained moderate and stable autophagic levels across the fifth instar. Subsequent untargeted metabolomics captured tissue- and stage-specific metabolic landscapes, among which lipids represented the most variable metabolite families. Clustering and KEGG enrichment analyses of differentially accumulated metabolites revealed conserved lipid divergence: fatty acid biosynthesis/metabolism pathways were enriched in high-autophagy tissues, whereas glycerolipids and glycerophospholipid metabolism dominated low-autophagy tissues. Integrative phenotypic and metabolomic evidence supports a close correlation between tissue-specific lipid remodeling and differential autophagic potency, implying that lipid metabolic rewiring may spatially modulate autophagy to drive metamorphic tissue remodeling. This work constructs a tissue-level dataset linking lipid metabolism and autophagy, offering a valuable framework for exploring lipid-autophagy crosstalk in insect metamorphosis; further functional validation is warranted to clarify their causal regulatory relationship.

## Data Availability

Data can be provided upon request to the corresponding author or the first author.

## Supplementary Materials

The following supporting information can be downloaded at https://www.mdpi.com/article/10.3390/insects17090949/s1, Table S1: Full list of all annotated metabolites with abundance data after merging and de-duplication of features acquired from positive and negative ionization modes. Figure S1: Gray value quantification of BmAtg8–PE protein bands of different tissues in *B. mori*. Figure S2: Immunofluorescent staining. Immunofluorescence analysis of various tissues across different developmental stages. Figure S3: LysoTracker Red staining of various tissues across different developmental stages. Figure S4: Tissue-specific metabolite clustering and overlapping differentially accumulated metabolites (DAMs) across *B. mori* larval tissues. Figures S5 and S6: Uncropped original western blot image corresponding to Figure S1.

## Abbreviations

Atg	Autophagy-related
PE	Phosphatidylethanolamine
Tubulin	Tubulin alpha 1a
5L2D	Day 2 of the 5th instar
5L3D	Day 3 of the 5th instar
5L4D	Day 4 of the 5th instar
5L5D	Day 5 of the 5th instar
5L6D	Day 6 of the 5th instar
5L7D	Day 7 of the 5th instar
W	The wandering stage
PP1	Day 1 of prepupa
PP2	Day 2 of prepupa
MTOR	Mechanistic target of rapamycin kinase
KEGG	Kyoto Encyclopedia of Genes and Genomes
20E:	20-Hydroxyecdysone
DAMs	differentially accumulated metabolites
